# Psychopathy, intelligence and emotional responding in a non-forensic sample: an experimental investigation

**DOI:** 10.1080/14789949.2014.943798

**Published:** 2014-08-12

**Authors:** Carolyn Bate, Daniel Boduszek, Katie Dhingra, Christopher Bale

**Affiliations:** ^a^ University of Huddersfield, West Yorkshire, UK

**Keywords:** psychopathy, intelligence, Levenson self-report psychopathy scale (LSRP), Raven’s Progressive Matrices IQ test

## Abstract

This study examined the relationships between psychopathy (primary and secondary), intelligence and emotional responding in a sample of 50 university students, using a task measuring autonomic responses to 40 pictorial stimuli (20 neutral and 20 emotionally provoking). Results indicated no significant direct relationship between primary or secondary psychopathy and emotional response, or primary or secondary psychopathy and intelligence. However, a significant moderating effect of intelligence on the association between both psychopathy factors and emotional response was observed, indicating those scoring higher on psychopathy but with lower intelligence portray the expected emotional responses to the affective stimuli (primary: *β *= −.56, *p *< .05; secondary: *β *= .80, *p *<* *.001). These findings indicate abnormal reactivity to emotional stimuli in lower intelligence, higher psychopathic individuals, and suggest differing roles for the two facets of psychopathy in affective responsiveness deviations.

## 1. Introduction

Psychopathy is characterised by a distinct cluster of interpersonal (e.g. deceitfulness and manipulation), affective (e.g. lack of empathy, remorse or guilt), and behavioural (e.g. irresponsibility and impulsivity) characteristics (Hare, 1996). The importance of psychopathy as a clinical construct has been demonstrated by multiple studies documenting a robust association between psychopathy and criminal behaviour (see Dhingra & Boduszek, 2013 for a review). Although clinical psychopathic samples have demonstrated reduced autonomic responses to emotional stimuli, no studies to date have measured autonomic responses to affective material in a non-clinical sample with psychopathic traits (Ali, Amorim, & Chamorro-Premuzic, 2009). Moreover, previous research has not considered the moderating role that intelligence may have on the association between psychopathy factors (primary and secondary) and emotional responses. This is an important omission as preliminary research indicates an interaction between psychopathy and intelligence may exist and that certain psychopathic individuals can control some physiological responses, similar to the manipulation of self-report scales, to give results that benefit themselves (Steinberg & Schwartz, 1975).

### 1.1. Psychopathy and intelligence

Early theorists such as Pinel (1801/1962) posited that a fundamental feature of psychopathy involved intact intellectual functioning in conjunction with antisocial tendencies. Cleckley (1941/1974), credited with the development of the first formalised criteria for psychopathy, suggested that the psychopath ‘is alert, usually more clever than the average person, and of a superior general objective intelligence, whether this is estimated by psychometric tests or by hearing him reason or talk’ (p. 240).

Previous research, however, has yet to convincingly demonstrate intellectual differences between psychopaths and non-psychopaths (Hare, 2003). Indeed, recent research on the relationships between intelligence measures and the Psychopathy Checklist Revised (PCL-R: Hare, 2003) indicates that the association is generally weak, and Hare and Neumann (2008) concluded that there is little reason to believe that psychopathic individuals possess superior intelligence. However, it is important to note that many studies have used total psychopathy rather than sub-scales scores. This is important given that recent studies suggest that the psychopathy factors are differentially related to external correlates (e.g. Dhingra, Boduszek, Palmer, & Shevlin, 2014). Consequently, it is possible that psychopathy factors could associate with intelligence scores in a number of different directions.

Consistent with the above proposition, Salekin, Neumann, Leistico, and Zalot (2004), using the Psychopathy Checklist: Youth Version (PCL:YV; Forth, Kosson, & Hare, 2003), found that the interpersonal dimension of psychopathy was positively associated with intelligence while the affective dimension was negatively associated with scores intelligence. In a replication and extension of Salekin et al.’s (2004) findings, Vitacco, Neumann, and Jackson (2005) found that verbal intelligence was positively related to the interpersonal dimension but negatively associated with affective and lifestyle dimensions. Finally, Neumann and Hare (2008) reported a pattern of results consistent with those of Vitacco et al. (2005). Thus, research to date suggests a pattern of differential relations between the psychopathy dimensions and various measures of intelligence, and indicates that the psychopathy–intelligence relationship may differ by sample type. These studies have, however, largely focused on samples of incarcerated males (Heinzen, Köhler, Godt, Geiger, & Huchzermeier, 2011), thus the true psychopathy–intelligence relationship may have been obscured.

Other researchers have suggested that an interaction exists between psychopathy and intelligence. However, empirical support for such an interaction is mixed. Heilbrun (1982) found that individuals with higher psychopathy scores and lower IQ scores had more previous violent and impulsive offences than those with high psychopathy and high IQ scores. Johansson and Kerr (2005) found that higher verbal intelligence scores among psychopathic individuals were associated with an earlier onset of criminal behaviour. This pattern was, however, reversed for non-psychopath criminals, for whom higher verbal intelligence served as a protective factor and postdicted later onset of criminal behaviour. By contrast, Walsh, Swogger, and Kosson (2009) found no interaction between psychopathy and intelligence in postdicting violence. Beggs and Grace (2008) found that offenders with relatively low intelligence and high psychopathy scores were more than four times likely than other offenders to recidivate sexually.

### 1.2. Psychopathy and emotional responding

Cleckley (1982) posited that psychopathic individuals do not develop appropriate morality because their early socialisation is not accompanied by normal affective experiences. Consistent with this, past research indicates that psychopathic individuals struggle to recognise emotions in others (Blair et al., 2004), have an inability to feel emotions themselves (Meffert, Gazzola, den Boer, Bartels, & Keysers, 2013; Visser, Bay, Cook, & Myburgh, 2010), demonstrate less differentiated emotional responses to distressing stimuli (Brook & Kosson, 2013; Patrick, 1994), experience difficulty in the processing or production of emotional language (Day & Wong, 1996), and exhibit behavioural psychophysiologic and regional brain activation anomalies when processing emotions (Brook & Kosson, 2013; Casey, Rogers, Burns, & Yiend, 2013; Osumi, Shimazaki, Imai, Sugiura, & Ohira, 2007). Furthermore, while non-psychopathic incarcerated adults show enhanced startle responses when viewing negative affective stimuli (i.e. images of assaults, mutilations and direct threat), incarcerated adults high on psychopathy show an attenuated response (Levenston, Patrick, Bradley, & Lang, 2000; Patrick, 1994; Patrick, Bradley, & Lang, 1993). Similarly, antisocial youth with psychopathic tendencies evidence reduced autonomic responses to distressing (i.e. crying child) and threatening (i.e. attacking dog) visual images (e.g. Blair, Jones, Clark, & Smith, 1997). Thus, psychopathic individuals evidence an absence of normal defensive reactivity (fear) in response to fearful or aversive stimuli.

Interestingly, results of a study by Levenston et al. (2000) suggest that psychopathic individuals react less than non-psychopathic individuals to both pleasant and unpleasant stimuli at a basic action–response level (e.g. electrodermal response, startle reflex modulation and electrocortical reactivity), despite normal verbal reports and overt facial expressions. This is consistent with the concept of dissociation between overt expressive behaviour and basic emotional response in psychopathy (Cleckley, 1982), and indicates the importance of using behavioural measures when examining emotional responding in psychopathy. Recent work also suggests that emotional responding may also be associated with the different factors of psychopathy. Kimonis, Frick, Fazekas and Loney (2006), for instance, found that the predicted association between psychopathic traits and reduced responsiveness to emotional stimuli only existed for those scoring highly on aggression traits. Similarly, Patrick, Cuthbert and Lang (1994) found greater attenuated autonomic activity among those scoring higher on the antisocial factor of psychopathy.

A limitation of the existing literature is that most studies have focused on the association between emotional responding and psychopathic traits in criminal samples (e.g. Loney, Frick, Clements, Ellis, & Kerlin, 2003; Patrick et al., 1993). This is surprising as, although research with non-clinical samples has demonstrated lower base rates of psychopathy, there is evidence for diverse expressions of psychopathic traits across the population (Skeem, Poythress, Edens, Lilienfeld, & Cale, 2003). The current study, therefore, seeks to extend the existing literature by investigating physiological reactions to emotional pictorial stimuli in a non-clinical sample.

### 1.3. The current research

Building on previous research (Brook & Kosson, 2013; Heinzen et al., 2011; Neumann & Hare, 2008; Salekin et al., 2004; Vitacco et al., 2005), the present study aims to investigate the direct relationships between psychopathy (two factors) and intelligence, and psychopathy (two factor) and emotional response, as well as the potential moderating role of intelligence in the psychopathy–emotional response relationship. Replicating links between psychopathy and IQ will provide additional credibility to Cleckley’s (1982) original hypotheses about psychopathy and intelligence. Additionally, examining the effect intelligence can have on the emotional response of those with high psychopathic tendencies will inform discussions of how highly intelligent psychopaths may remain undetected within society.

## 2. Method

### 2.1. Participants

Participants were 50 undergraduate students who participated in the experiment in exchange for course credits. Their ages ranged from 18 to 41 years (*M *= 22.64, SD =* *6.49). Forty-three were White British (86%), 1 Bulgarian (2%), 1 Indian British (2%), 1 Lithuanian (2%), 1 African British (2%), 1 South African (2%), 1 Polish (2%) and 1 Romanian (2%). Participants all had normal, or corrected to normal vision.

### 2.2. Materials

*Levenson self-report psychopathy scale* (*LSRP; Levenson, Kiehl, & Fitzpatrick,* *1995*). The LSRP is a 26-item self-report measure designed to assess psychopathic traits in non-institutionalised samples. The primary psychopathy scale consists of 16 items, designed to assess the core personality features described by Cleckley (1982), such as being selfish, uncaring and manipulative. The secondary psychopathy scale consists of 10 items assessing antisocial behaviour, a self-defeating lifestyle and impulsivity. Items are rated in the range of 1 = *disagree strongly* to 4 = *agree strongly scale.* Cronbach’s *α* in the current study were .82 for the primary psychopathy scale and .74 for the secondary psychopathy scale.

*The Raven’s Standard Progressive Matrices* (*Raven,* *1938*) are a standardised measure for intelligence. The test consists of 60 problems split into five sets of 12 of increasing difficulty, with each prior set providing training for later sets. An individual’s total score provides an indication of intelligence and the scale has been found to have a test–retest reliability varying between .83 and .93 (Raven, 1938) and a significant strong positive correlation to other known validated IQ tests.

*Image task using The International Affective Picture System* (*IAPS; Lang, Bradley, & Cuthbert,* *1997*). To create the image set we selected 40 pictorial stimuli from the IAPS, 20 neutral (IAPS numbers: 2190, 2200, 2210, 2230, 2381, 2440, 2480, 2570, 2850, 7002, 7009, 7010, 7020, 7030, 7040, 7080, 7175, 7233, 7235 and 9070) and 20 emotionally provoking (IAPS numbers: 1050, 1120, 1201, 1300, 1930, 3000, 3010, 3050, 3060, 3071, 3080, 3102, 3110, 3130, 3530, 6260, 6350, 6510, 6540 and 9405), based on pleasure and arousal ratings. The IAPS is a database containing over 1000 standardised, emotionally evocative, internationally accessible, colour photographs, portraying a wide range of semantic categories. The emotionally provoking pictures used were not bloody, depicting graphic violence or pornographic in nature. They were chosen to mildly shock the participant or create an empathetic response. Normative ratings of valence for pictures in these categories differed (neutral: 4.9, unpleasant: 2.6) as did normative ratings of arousal (pleasant: 5.7, neutral: 2.6; unpleasant: 6.4; Lang, Bradley, & Cuthbert, 1999). Pictures were displayed for two seconds each with a rest period of two seconds in between each stimulus, timings based on prior research (Lithari et al., 2010).

*A Biopac MP35 Acquisition Unit* (Biopac Systems Inc., 2013) and galvanic skin response (GSR) electrodes with sigma gel were used to record physiological responses to the pictorial stimuli onto the Biopac Student Lab Pro software.

### 2.3. Procedure

All participants were tested individually in a quiet university room. Each participant was seated in a comfortable chair in front of a computer on which the stimuli were to be displayed. Participants completed the questionnaire measure and were then attached to the Biopac through electrodes on their fore and middle fingers. GSR was then recorded for 60 s, to provide a baseline, before being presented with each image sequentially. The 40 images were displayed in a randomised order for each participant, each for two seconds with a two second target screen between each image to avoid interference of the previous stimuli.

The activity of the sweat glands in response to sympathetic nervous stimulation results in an increase in the level of skin conductance. BIOPAC software calculates SCL/SCR in μmho, the traditional unit of conductance. Micromho (μmho) is interchangeable with the alternative microsiemens (μS). GSR was calculated as an average of all GSR values across all picture presentations, and all participants. Although no limits were issued for the images, all participants were asked to look carefully at each image.

## 3. Results

### 3.1. Descriptive statistics

Descriptive statistics for the two factors of psychopathy, age, intelligence, emotional response and age, including means (*M*) and standard deviations (SD), are presented in Table 1.

**Table 1.** Descriptive statistics for psychopathy, intelligence and emotional response.

	IQ	B	ER	ER-B	F1	F2
*M*	49.52	8.88	8.63	−.24	34.92	22.16
SD	5.32	4.80	4.18	1.52	9.36	5.95
Range	24.00	28.79	21.46	12.02	46.00	31.00
Minimum	34.00	.67	.78	−7.21	19.00	9.00
Maximum	58.00	29.46	22.25	4.81	65.00	40.00

Note: IQ = intelligence; B = baseline GSR reading; ER = emotional response GSR reading; ER-B = emotional response minus baseline GSR reading; F1 = psychopathy factor 1; F2 = psychopathy factor 2.

### 3.2. Correlations between intelligence, psychopathy and emotional responses

Intercorrelations among all variables were investigated using Pearson product-moment correlation coefficients (Table 2). Results indicated no significant associations between either of the psychopathy factors and intelligence, or either of the psychopathy factors and emotional responses. Moreover, no direct relationship between intelligence and emotional response to affective images was found. A weak moderate negative correlation was found between age and psychopathy factors 1 (*r *= .33) indicating that as age increases, the interpersonal and affective traits of psychopathy decrease.

**Table 2.** Correlations between psychopathy, IQ, emotional response and age.

Variables	F1	F2	IQ	ER	Age
F1	–				
F2	.50^***^	–			
IQ	.13	−.01	–		
ER	−.09	.20	−.09	–	
Age	−.33^*^	−.19	−.05	.23	–

Notes: IQ = intelligence; ER = emotional response; F1 = psychopathy factor 1; F2 = psychopathy factor 2.

^*^ *p *<* *.05

^***^ *p *<* *.001.

### 3.3. Moderation regression analysis

Hierarchical moderated regression analysis was performed to investigate the ability of psychopathy to predict emotional response to affective images, whilst controlling for IQ. Preliminary analysis ensured no violation of the assumptions of normality, linearity and homoscedasticity.

In the first step of the hierarchical moderated multiple regression, the main effects of psychopathy factors one and two and IQ on emotional response were investigated. This model (model 1) was not statistically significant *F*(3, 46) = 1.54, *p *> .05 and explained three per cent of variance in emotional response (Adj *R* ^2^ = .03). None of the predictor variables significantly contributed to the prediction of emotional responses to stimuli (Table 3).

**Table 3.** Regression model of the association between psychopathy factors and emotional response with IQ as a moderating factor.

	*R*^2^	Adj *R*^2^	*β*	SE
Model 1	.09	.03		
F1			−.24	.16
F2			.32	.16
IQ			−.06	.14
Model 2	.31	.19		
F1			−.17	.18
F2			.27	.16
IQ			.01	.15
F1 by IQ (1SD above mean)			.22	.25
F1 by IQ (mean)			−.17	.20
F1 by IQ (1SD below mean)			−.56^*^	.25
F2 by IQ (1SD above mean)			−.27	.25
F2 by IQ (mean)			.27	.23
F2 by IQ (1SD below mean)			.80^***^	.21
Age			.01	.02
Gender			.09	.43

Notes: IQ = intelligence; F1 = psychopathy factor 1; F2 = psychopathy factor 2.

^*^ *p *< .05

^***^ *p *< .001.

The second step consisted of entering interaction terms, coding interaction between IQ and psychopathy. Incorporation of the interactions explained an additional 16% of the variance and the final regression model (model 2) explained 19% of variance in emotional response (Adj *R* ^2^ = .19), *F*(7, 42) = 2.65, *p *< .05. There were no significant direct relationship between predictor variables and emotional responses. However, for both psychopathy factors, the results indicate that a significant association occurs only at low levels (−1 SD) of intelligence. For psychopathy factor one, at a low intelligence level, a significant strong negative association is observed between psychopathy and emotional response (*β *= −.56), indicating that as factor one scores increases, emotional response decreases. For psychopathy factor two, at a low intelligence level, a significant strong positive association is observed between psychopathy and emotional response (*β *=* *.80), indicating as factor two scores increase, so does emotional response.

## 4. Discussion

The present study aimed to examine whether non-clinical psychopathy (primary and secondary) is associated with emotional responding and intelligence, and whether the psychopathy–emotional responding relationship is moderated by intelligence. The predicted association between psychopathy and emotional responsiveness was only found for individuals with lower intelligence.

Our finding of a differential relation between the two psychopathy dimensions, intelligence and emotional responding may help to understand why the literature is mixed on the topic of associations between psychopathy and intelligence and supports previous research (e.g. Dhingra, Boduszek, Hyland, & Debowska, 2014) suggesting that the dimensions of psychopathy may be differentially related to various external correlates. Specifically, our analysis demonstrated that for individuals with lower levels of intelligence, there is a negative association between factor one psychopathy scores and emotional responses to evocative images, and this accords with previous research demonstrating a relative lack of emotional responsiveness, as measured by GSR, in individuals scoring highly on psychopathy (Lorber, 2004). Factor one consists of the interpersonal and affective facets of psychopathy, which subsume traits relating to callousness, lack of empathy and the manipulation of others, and these core traits are argued to be reflected in deficits in emotional processing (Brook, Brieman, & Kosson, 2013). However, for participants with mean and higher levels of intelligence there was no association between psychopathy and emotional responsiveness. It may be the case that higher levels of intelligence facilitate the regulation of emotional responses in individuals with high levels of psychopathy. Given that psychopaths have been demonstrated to be capable of regulating their GSRs (Steinberg & Schwartz, 1975), these individuals may understand and be able to reproduce normative physiological responses to evocative stimuli, which could facilitate their remaining undetected in wider society. Thus, our findings may have implications for understanding the phenomenon of *corporate psychopaths* (Boddy, Ladyshewsky, & Galvin, 2010).

Our results also demonstrate that for individuals with lower levels of intelligence, there is a positive association between factor two psychopathy scores and emotional responsiveness. Given that previous research has typically demonstrated negative relationships between psychopathy and emotional response (Lorber, 2004), this was an unexpected finding. Factor two encapsulates the behavioural characteristics of psychopathy, including impulsivity, poor planning and control of behaviour, and delinquency. We suggest that individuals with lower levels of intelligence and higher levels of factor two psychopathy may be especially aroused by negative emotionally evocative stimuli, but that rather than being aversive, they may experience this as fulfilling a greater need for stimulation. Previous research indicates that baseline levels of electrodermal activity are lower for individuals who are high in psychopathy (Lorber, 2004), and low arousal is an aversive state that can be compensated for by sensation seeking and risk taking (Zukerman, 1974). Thus, individuals with high levels of factor two psychopathy and low levels of intelligence may seek out highly stimulating contexts in order to compensate for the reduced internal stimulation they typically experience, and such sensation seeking may lead to engagement in criminal behaviour (Raine, Reynolds, Venebales, Mednick, & Farrington, 1998). In contrast, high factor two scoring individuals with higher levels of intelligence may be more capable of suppressing this arousal from evocative stimuli, which could facilitate more normative behaviour and help them to avoid impulsive or antisocial behaviour which could lead to incarceration. Alternatively, individuals with lower intelligence and higher factor two psychopathy may have been reacting with increased negative affect to the emotionally arousing stimuli (i.e. a defensive response to promote withdrawal or avoidance behaviour in the presence of aversive cues). This would be consistent with research by Patrick (1994) which reported that measures of emotional distress and fear were negatively related to PCL-R Factor 1 scores after controlling for PCL-R Factor 2 and positively related to Factor 2 after controlling for Factor 1 (see also Hicks & Patrick, 2006). Thus, further research is needed to determine whether this response is either an appetitive or defensive.

We note that the majority of previous research on the relationship between psychopathy and emotional responsiveness has been conducted on incarcerated samples (Brook et al., 2013). Given that lower IQ scores predict more criminal behaviour (Levine, 2011), individuals with lower levels of intelligence are likely to be over-represented in these samples. This may explain why previous research typically demonstrates an association between psychopathy and emotional responsiveness (Lorber, 2004), where our results show no overall relationship (with an association only in participants with lower levels of intelligence). These results suggest that future research on the relationship between psychopathy and emotional responsiveness should include measures of intelligence, and control for this in analyses of overall relationships.

Although there has been little previous research on emotional regulation in psychopathy, a recent study by Casey et al. (2013) demonstrated that in a sample of violent offenders, individuals with higher levels of factor one psychopathy were less able to regulate their emotional responses (increasing their reactivity to negative images) when instructed to attempt to consider them empathically. Future studies could employ such emotion regulation paradigms to examine whether intelligence moderates this relationship between psychopathy and emotional response. Results demonstrating that more highly intelligent psychopathic individuals are more able to produce normative empathic responses when employing these paradigms would support our suggestion that higher levels of intelligence may facilitate emotional regulation in psychopaths. Such findings would also have important implications for the treatment and rehabilitation of psychopathic offenders. Our results suggest that clinicians should consider the intelligence of psychopathic individuals when developing and administering interventions as individuals higher in intelligence may be better able to present a façade of change (i.e. expressing regret and victim empathy) and compliance to treatment, and thus able to re-enter society and recidivate. This is consistent with research indicating that higher PCL-R scores are associated with lower scores on global measures of clinical change (Hughes, Hogue, Hollin, & Champion, 1997).

The use of a small, undergraduate sample in our study may limit generalisability to other non-clinical groups. However, undergraduate samples have the advantage of being relatively free of severe Axis I disorders, which could impact upon the accurate reporting of personality traits (Lilienfeld & Penna, 2001). Despite this limitation, the current results suggest that even in a small student sample, intelligence may moderate the relationship between psychopathy and emotional processing and response.

Our results demonstrate no relationships between either factor one or two psychopathy and intelligence in our student sample, supporting previous research on incarcerated samples (Hare, 2003). Our analyses suggest that instead of demonstrating a direct relationship with psychopathy, intelligence may instead moderate relationships between psychopathic traits and associated variables (e.g. violent and impulsive crime; Heilbrun, 1982), and thus future research on such relationships should include, and potentially control for, measures of intelligence.

The current study could be extended to include both visual and acoustic affective stimuli in order to assess whether our findings generalise across different stimulus modalities. It would also be profitable to include other physiological measures (e.g. heart rate) which have shown inconsistent results in comparison to GSR measures in previous research on psychopathy and emotional response (Brook et al., 2013; Lorber, 2004). Similarly, it would be interesting to examine the effects of stimulus valance to assess whether more highly intelligent highly psychopathic individuals also demonstrate more normative emotional responses to positive stimuli.

In conclusion, the current study found no direct relationships between psychopathy, intelligence and emotional responsiveness in our student sample. Instead, our results demonstrate that intelligence moderates the relationship between psychopathy and emotional response, but that the nature of this interaction differs between primary and secondary psychopathy. We encourage researchers to include measures of intelligence in future studies, in order to examine whether it also moderates other relationships between psychopathy and related constructs. In addition, researchers should conduct separate analyses of the two factors of psychopathy, rather than treating it as a unitary construct. Developing a greater understanding of the complex relationships between psychopathy, intelligence and related constructs will have important implications, both in clinical, and wider social settings.
